# On the Evolution of Light from the Living Human Subject

**Published:** 1842-08-13

**Authors:** 


### ANALECTA.

*On the Evolution of Light from the Living Human Subject.*—Sir Henry Marsh, Bart. M. D. appears in a long communication under the above head, in the Provincial Medical Journal for June 4th, 1842. The paper commences with a detail of many phenomena resulting from the decomposition of organic substances capable of evolving light, and from the elimination of this subtile agent during life, by certain vegetables and many of the inferior animals. The seremarks are interesting, but present little that is novel; and we therefore pass them by without further notice, and proceed to make a few short extracts from cases of somewhat similar appearances in the human subject found towards the conclusion of the article.

The following account is quoted by Sir Henry as resting upon the authentic and unquestionable authority of a friend—

"It was ten days previous to L. A.'s death that I first observed a very extraordinary light, which seemed darting about the face, and illuminating all around her head, flashing very much like an aurora borealis. She was in a deep decline, and had that day been seized with suffocation, which teazed her much for an hour, and made her so nervous that she would not suffer me to leave her for a moment, that I might raise her up quickly in case of a return of this painful sensation. After she settled for the night I lay down beside her, and it was then this luminous appearance suddenly commenced. Her maid was sitting up beside the bed, and I whispered to her to shade the light, as it would awaken Louisa. She told me the light was perfectly shaded. I then said, 'What can this light be which is flashing on Miss Louisa's face?' The maid looked very mysterious, and informed me she had seen that light before, and it was from no candle. I then inquired when she had perceived it; she said that morning, and it had dazzled her eyes, but she had said nothing about it, as ladies always considered servants superstitious. However, after watching it myself half an hour, I got up and saw that the candle was in a position from which this peculiar light could not have come, nor, indeed, was it like that sort of light; it was more silvery, like the reflection of moonlight on water. I watched it for more than an hour, when it disappeared. It gave the face the look of being *painted white* and *highly glazed*, but it danced about and had a very extraordinary effect. Three nights after, the maid being ill, I sat up all night, and again I saw this luminous appearance, when there was no candle nor moon, nor, in fact, any visible means of producing it. Her sister came into the room and saw it also. The evening before L. A. died I saw the light again, but it was fainter, and lasted but about twenty minutes. The state of body of the patient was that of extreme exhaustion. For two months she had never even sat up in bed. Many of her symptoms varied much from those of other sufferers in pulmonary complaints whom I had seen, but the general outline was the same. Her breath had a very peculiar smell, which made me suppose there might be some decomposition going forward."

The fact is certain, the source whence derived authentic, the circumstantial detail clear and conclusive. The person from whom I derived the knowledge of this interesting fact is one of a clear head, superior power of observation, and utterly exempt from the distortions and exaggerations of superstition. The young lady about whose person these luminous appearances were manifested, I had seen several times before her return to the country; her lungs were extensively diseased; she laboured under the most hopeless form of pulmonary consumption.

Sir Henry then quotes the following statement made to him by the sister of one of his deceased patients :—

" About an hour and a half before my dear sister's death, we were struck by a luminous appearance proceeding from her head in a diagonal direction. She was at the time in a half recumbent position, and perfectly tranquil. The light was pale as the moon, but quite evident to mamma, myself, and sister, who were watching over her at the time. One of us at first thought it was lightning, till shortly after we fancied we perceived a sort of tremulous glimmer playing round the head of the bed ; and then recollecting we had read something of a similar nature having been observed previous to dissolution, we had candles brought into the room, fearing our dear sister would perceive it, and that it might disturb the tranquillity of her last moments."

The prior of the two following cases appears to have produced considerable excitement at the time, in the neighbourhood where it occurerd. After some prefatory remarks, the writer proceeds—

An authentic report of this interesting phenomenon having been subsequently published by Dr. Donovan in the "Dublin Medical Press," Jan. 15, 1840, I shall extract from his statement the most important particulars of the case. The name of the patient was Harrington, and he resided in a small but populous village on the harbour of Glandore, one of the most picturesque spots on the southern coast of Ireland. Dr. Donovan states, " I was sent for in December, 1828, to see Harrington ; he had been under the care of my predecessor and had been entered in the dispensary book as a phthisical patient, and on reference to my note-book, I find that the stethoscopic and other indications of phthisis were indubitable. He was under my care for about five years, during which time, strange to say, the symptoms continued stationary, and I had discontinued my attendance for about two years when the report became general that mysterious lights were every night seen in his cabin. The subject attracted a great deal of attention, and, like every thing else in Ireland, at once assumed a sectarian complexion ; some attributing the lights to the miraculous interposition of heaven, others to the practice of the black art. Not regarding these views as offering any explanation of the mystery, I determined to subject the matter to the ordeal of my own senses, and for this purpose visited the cabin for fourteen nights, and on three nights only did I witness any thing unusual ; once I perceived a luminous fog resembling the aurora borealis ; and twice I saw scintillations, like the sparkling phosphorescence sometimes exhibited by the sea infusoria. From the close scrutiny I made, I can with certainty say that no imposition was either employed or attempted." Dr. Donovan adds, " I am of opinion that the appearances which I witnessed were dependant on the presence of phosphorescent matter in the expiratory and perspiratory secretions. The property which phosphuretted hydrogen has of undergoing spontaneous ignition when brought into contact with atmospheric air is well known ; and as the components of which this is made up exist in abundance in the human body, it is not outstretching the bounds of probability to suppose that it is sometimes generated in the living system."

Dr. Donovan has lately informed me that the luminous appearances were not any time visible to him on the person of the patient ; the scintillations which he has described above were perceptible immediately over the head of his bed on a wall composed of stone and clay mortar. The luminous fogs passed in streams through the apartment in which the sick man lay, and on one occasion Dr. Donovan fancied that he saw a meteor, like a falling star, suddenly pass through the house. Appearances of a similar nature are said to have been seen about the person of a woman named Pallister who died some time ago in the neighbourhood of Hull; but I have not been able to procure any well authenticated report of the phenomena.

For the following very interesting and important fact, I am indebted to my friend Dr. William Stokes:—"When I was residing in the Old Meath Hospital, a poor woman labouring under an enormous cancer of the breast was admitted. The breast was much enlarged, and presented a vast ulcer with irregular and everted edges, from all parts of which a quantity of luminous fluid was constantly poured out. Upon being asked whether she suffered much pain, she answered, "Not now, sir, but I cannot sleep watching this sore which is on fire every night." I directed that she should send for me whenever she perceived the luminous appearance, and on that night I was summoned between ten and eleven o'clock. The lights in the ward having been then extinguished, she was sitting leaning forward, the left hand supporting the tumour, while with the right she every now and then lifted up the coverings of the ulcer to gaze on this, to her, supernatural appearance. The whole of the base and edges of the cavity phosphoresced in the strongest manner. The light was steady. I directed that the ulcer should remain open, while I retreated gradually, keeping my eyes fixed on the light, which I found distinctly visible at the end of the ward, which must have been more than twenty feet from her bed. The light within a few inches of the ulcer was sufficient to enable me to distinguish the figures on a watch dial. I have no very distinct recollection of the colour of the light, but I remember that its intensity was variable, it being on some nights much stronger than on others."

After a few chemico-physiological conjectures about these cases Sir Henry adds—

Between the facts now recorded, and the state of the body when *spontaneous combustion* takes place, there is a very striking analogy. In one case of spontaneous combustion which is related on good authority, from the person who suffered from this mysterious and, as we may justly call it dreadful, disease, a lambent flame was distinctly seen to issue; this was observed while the patient was still alive, for he lived for four days after the occurrence of the spontaneous combustion, and was able to give an accurate account of his earliest sensations. This is an important fact, and bears directly on the subject we are discussing; before internal combustion can take place, the vital property must have nearly lost its controlling influence, as already explained, and chemical action, before death, must have begun to predominate. This explanation is corroborated by another case, the details of which have been published. It is the fact of the disengagement of a gas throughout every part of the body, which became universally emphysematous; on making an incision into any part the gas escaped, which, when touched with the flame of a candle, ignited and burned with a blueish flame.

The paper concludes with the following experiment by M. Magendie—

Half an ounce of olive oil, in which two grains of phosphorus were dissolved, was injected into the crural vein of a dog. Before the syringe was completely emptied, a dense white vapour began to issue from the nostrils, which became faintly luminous on the removal of the lights. An additional half ounce of phosphorated oil, of equal strength, was then injected, and the lights extinguished. The expirations immediately became beautifully luminous, resembling jets of pale coloured flame pouring forth from the nostrils of the animal. This extraordinary spectacle continued until the death of the dog, which occurred in about five minutes. In this experiment the rapid transit of the injected fluid from the vein in the remote extremity to the lungs, and its instantaneous expiration in the gaseous form, were very remarkable. The result of the experiment would also seem to show that in some, at least, of the instances of luminous appearances referred to, the presence of phosphorus formed an element in the production of the effect.

